# The effect of FK506 (tacrolimus) loaded with collagen membrane and fibrin glue on promotion of nerve regeneration in a rat sciatic nerve traction injury model

**DOI:** 10.1186/s40902-022-00339-5

**Published:** 2022-04-06

**Authors:** Jin-Hong Kim, Young-Jin Choi, Han-Ick Park, Kang-Min Ahn

**Affiliations:** Department of Oral and Maxillofacial Surgery, Asan Medical Center, College of Medicine, University of Ulsan, 88, Olympic-ro 43-gil, Songpa-gu, Seoul, 05505 Korea

**Keywords:** Sciatic nerve, Traction injury, Nerve regeneration, FK506, Collagen, Fibrin glue

## Abstract

**Background:**

Peripheral nerve injury is one of the most common injuries that might occur in oral and maxillofacial surgery. The purpose of this study was to determine the effect of FK506 loaded with collagen membrane and fibrin glue on the promotion of nerve regeneration after traction nerve injury in a rat model.

**Methods:**

Thirty male Sprague-Dawley rats were divided into three groups: group A (*n* = 10), a sham group whose sciatic nerve was exposed without any injury; and groups B (*n* = 10) and C (*n* = 10), which underwent traction nerve injury using 200 g of traction force for 1 min. The injured nerve in group C was covered with a collagen membrane soaked with FK506 (0.5 mg/0.1 mL) and fibrin glue. Functional analysis and microscopic evaluation were performed at 2 and 4 weeks after injury.

**Results:**

The sciatic function index was − 5.78 ± 3.07 for group A, − 20.69 ± 5.22 for group B, and − 12.01 ± 4.20 for group C at 2 weeks after injury. However, at 4 weeks, the sciatic function index was − 5.58 ± 2.45 for group A, − 19.69 ± 4.81 for group B, and − 11.95 ± 1.94 for group C. In both periods, statistically significant differences were found among the groups (*p*<0.017). Histomorphometric evaluation revealed improved nerve regeneration in group C compared to that in group B. However, no statistical differences in axonal density were found among the three groups (*p* < 0.017).

**Conclusion:**

Localized FK506 with collagen membrane and fibrin glue could promote axonal regeneration in a rat model of traction nerve injury.

## Background

Peripheral nerve injuries (PNIs) might occur during operation and could lead to loss of function or chronic pain [1]. Stretch-related injuries (traction), laceration, and compression are the three basic types of PNI [2]. Traction nerve injury (TNI) is one of the most common traumatic injuries that might occur in oral and maxillofacial surgeries. Injuries of this type can occur in third molar extraction, orthognathic surgery, fracture reduction, parotidectomy, temporomandibular joint surgery, oral cancer surgery, and free flap reconstruction [3–8]. Most TNIs result in transient dysfunction; however, some might develop as permanent anesthesia, paresthesia, or motor function loss [9]. Establishing clinically applicable techniques for the treatment of TNIs is thus essential.

Many attempts have been made to treat PNI. Several methods have been proposed, such as conduits (e.g., autogenous, nondegradable, or degradable materials) [10–12], cell-based therapy (e.g., Schwann cells, olfactory ensheathing cells, bone marrow–derived mesenchymal stem cells, and pluripotent stem cells) [13–16], and growth factors (e.g., nerve growth factor, neurotrophin-3, and basic fibroblast growth factor) [17–19]. Although these developments showed promising results, clinical limitations, including cost, ethics, and preparation time limitations, were found.

The immunosuppressant drug, FK506 (tacrolimus), prevents allograft rejection, which is crucial for organ transplantation. FK506 has similar effects to cyclosporine A (CsA) as it inhibits the transcription of early T cell activation genes, apparently by modulating the activity of transcriptional regulators [20]. Another characteristic of FK506 is its neuroregenerative effect. Gold et al. [21] first reported that FK506 promotes nerve growth in vivo in a rat model of crush nerve hind limb. In vitro, FK506 promotes neurite outgrowth by increasing sensitivity to nerve growth factor [22]. Moreover, the systemic administration of FK506 increases nerve regeneration in rat PNI in a dose-dependent manner [23, 24]. Other studies reported significant results with topical application but with lesser systemic toxicity. Diaz et al. [25] achieved facial nerve regeneration by applying FK506 topically in a rabbit model using entubulation neurorrhaphy. Yeh et al. [26] demonstrated similar results regarding facial nerve crush injury using a rat model. Tajdaran et al. [27] reported enhanced axon regeneration in rats with different types of local delivery systems. However, the effect of FK506 on TNIs has not yet been determined.

The purpose of our study was to verify the neurotrophic effect of localized FK506 on TNI in a rat model. Functional analysis and histomorphometric examination were conducted at 2- and 4-week intervals after surgery to assess nerve regeneration.

## Materials and methods

This experiment was approved by the ethics committee on the experimental use of animals of the Animal Research Committee (2021-11-210).

### Preparation of FK506 (tacrolimus)

**Fig. 1:**
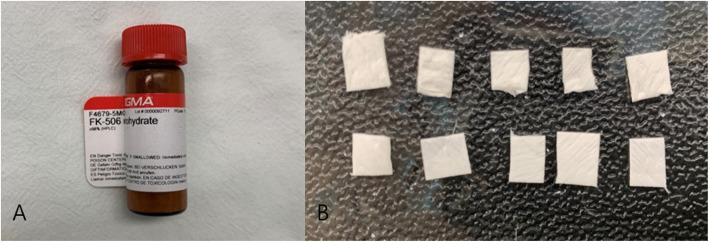
Preparation of FK506. **A** FK506 monohydrate. **B** Collagen membrane loaded with FK506

FK506 (F4679, Sigma-Aldrich, St. Louis, USA), supplied as a powder (5 mg) in a glass bottle, was used in this study. The powder was dissolved with 1 mL of normal saline (20 mL, Choongwae Pharm, Korea) before application. The concentration of FK506 (0.5 mg/0.1 mL) was selected based on previous published studies [27, 28]. Collagen membrane (OSSGUIDE®, Bioland Co., Cheongju, Korea, 15 mm × 20 mm × 0.2 mm) was cut into 10 pieces, with each piece having a size of 5 mm × 5 mm × 0.2 mm. The membranes (*n* = 10) were hydrated with 0.1 mL of FK506 solution (0.5 mg/0.1 mL) (Fig. 1).

### Surgical procedures

Thirty male Sprague-Dawley (SD) rats (weight, 250 g) were divided into three groups (*n* = 10 for each group) and anesthetized via an intraperitoneal administration of 30 mg/kg of Alfaxan (Jurox, Rutherford, Australia) and 10 mg/kg of Rompun (Bayer, Leverkusen, Germany). After routine povidone-iodine (Betadine™, Choongwae Pharm, Korea) preparation of the operative field on the left thigh, all rat limbs were fixed on the experimental plate with a plaster. The left sciatic nerve was exposed through a 1.5-cm straight skin incision at the posterior surface of the upper thigh. After intermuscular fascial dissection, approximately 10 mm of the sciatic nerve was completely isolated and dissected from the adjacent tissue and muscle.

**Fig. 2:**
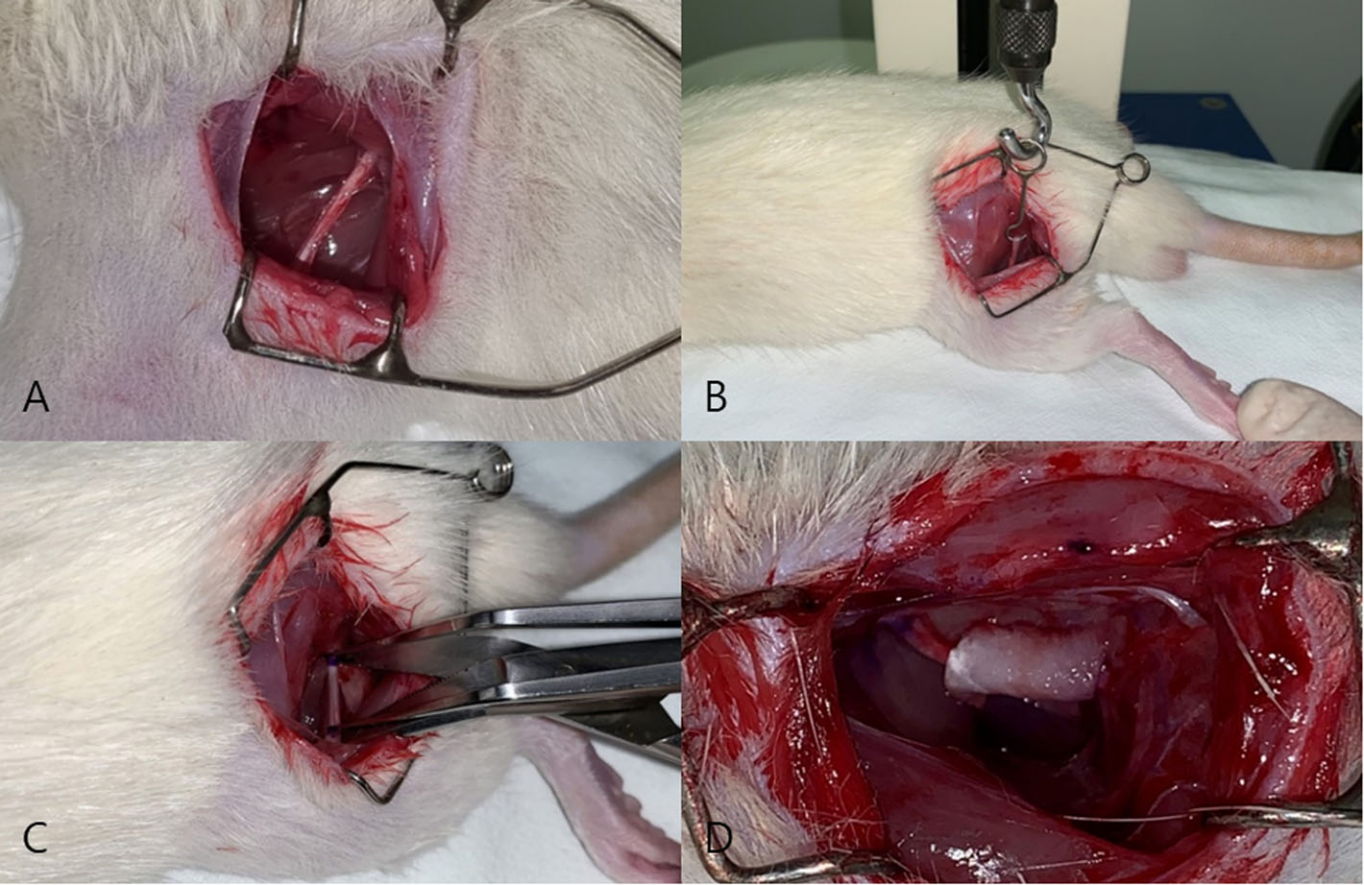
Surgical procedures. **A** Exposure of the sciatic nerve of a rat. **B** Precise traction injury performed in the left sciatic nerve of the rat. **C** Measurement of the elongated sciatic nerve after traction injury. **D** Application of FK506 loaded with collagen membrane and fibrin glue on the injured sciatic nerve of a rat

Rats in group A underwent sham operation with no injury of the sciatic nerve, while rats in groups B and C underwent traction injury using 200 g of traction force for 1 min. Before traction injury, both ends of the tension site equivalent to 10 mm were marked with an indelible pencil. The final tension force and the sciatic nerve length were measured to compare the amount of change after traction injury. In group C, collagen membrane loaded with FK506 was used to cover the dissected nerve. Fibrin glue (Beriplast P, CSL Behring, Germany) was loaded after this application. The skin was closed with 4-0 Vicryl™ (Ethicon, UK) via the layered suture technique. Antibiotic (Amoxicillin™ 150 mg/kg SC, Il Sung Pharm, Korea) and analgesic (Ketorolac™ 2 mg/kg SC, Dong Kook Pharm, Korea) were injected intramuscularly after operation every 24 h for 1 week. The surgical procedure was identical for all animals (Fig. 2). All rats were supplied with food and water ad libitum.

### Functional analysis

**Fig. 3:**
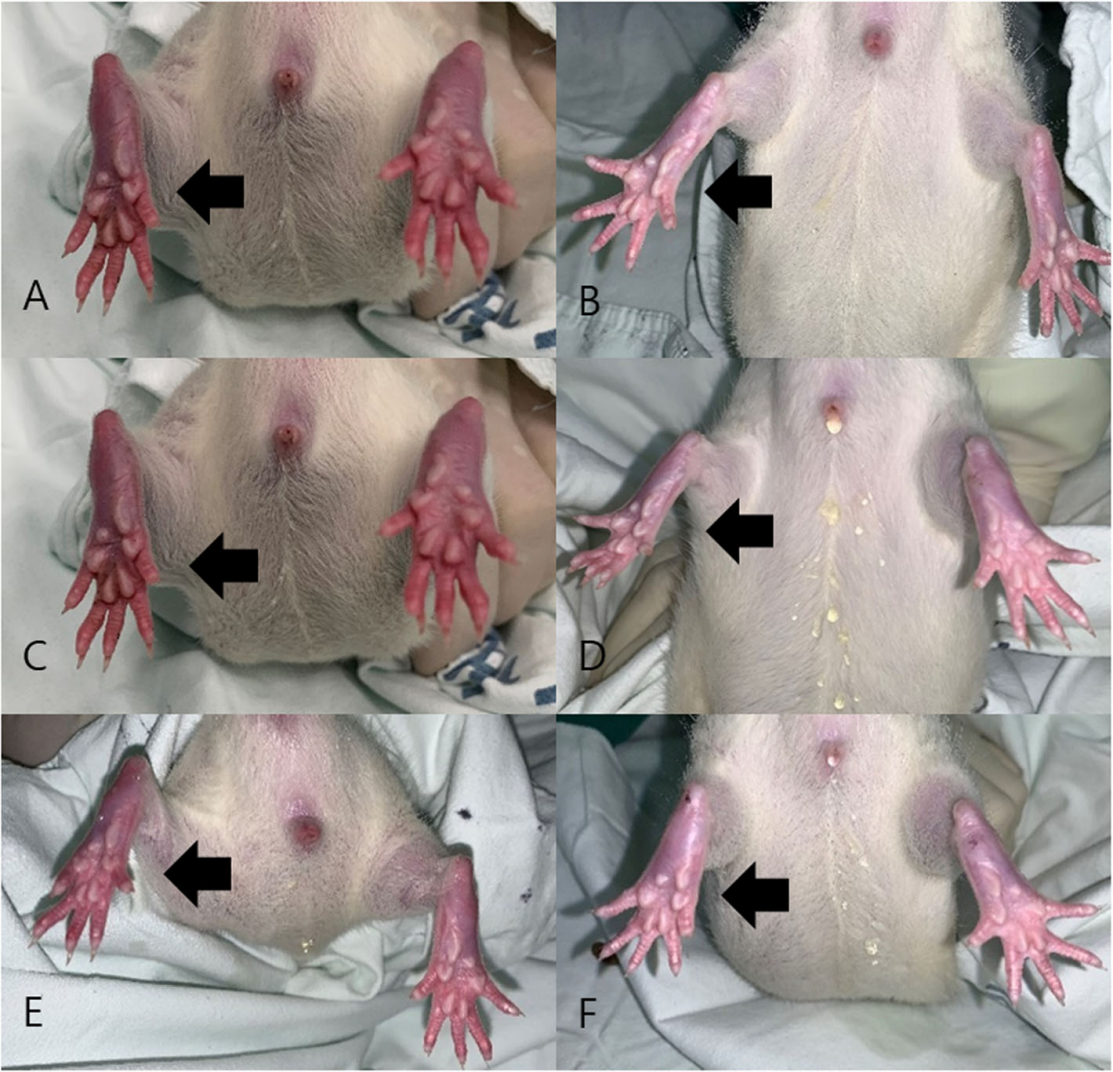
Images of the sole. **A** Representative of group A at 2 weeks. **B** Representative of group A at 4 weeks. **C** Representative of group B at 2 weeks. **D** Representative of group B at 4 weeks. **E** Representative of group C at 2 weeks. **F** Representative of group C at 4 weeks. Both feet are spread evenly (arrow) in panels **A** and **B**. The left foot appears crooked compared to the right foot (arrow) in panels **C** and **D**. The left foot appears crooked compared to the right foot in panel **E**; however, the left foot appears to have recovered slightly (arrow) in panel **F**

**Fig. 4:**
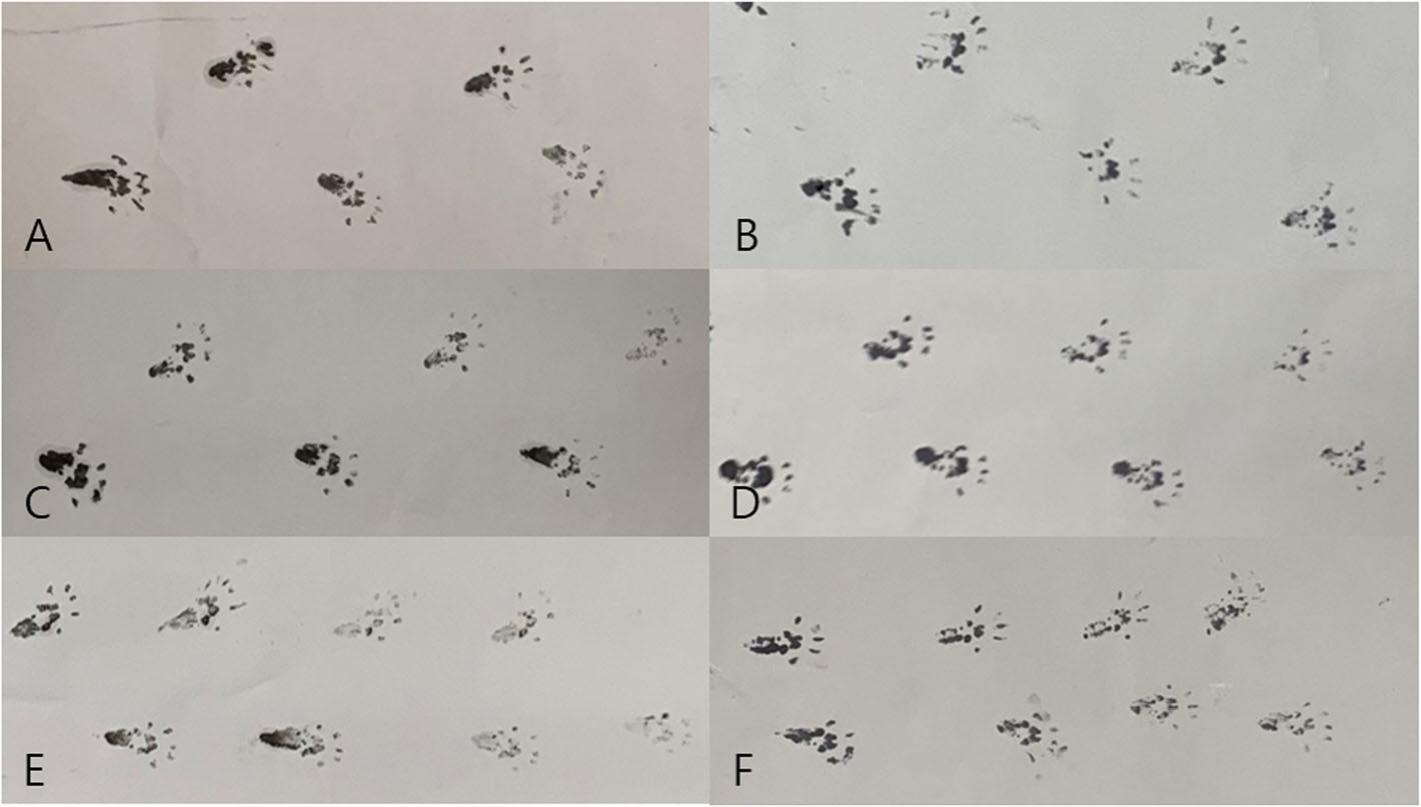
Walking track analysis. **A** Footprints of group A at 2 weeks. **B** Footprints of group A at 4 weeks. **C** Footprints of group B at 2 weeks. **D** Footprints of group B at 4 weeks. **E** Footprints of group C at 2 weeks. **F** Footprints of group C at 4 weeks

The function of the sciatic nerve was evaluated 2 and 4 weeks after operation. Each week, five rats in each group were randomly selected for testing. For each evaluation, a walking track analysis was performed and the sciatic function index (SFI) value was calculated. Footprints were recorded on a white paper when rats walked on a corridor. The following parameters were measured from the footprints: (1) distance from the heel to the top of the third toe (print length; PL), (2) distance between the first and fifth toes (toe spreading; TS), and (3) distance from the second to fourth toes (intermediary toe spreading; IT). These measures were recorded from the experimental sides (EPL, ETS, and EIT) and the nonoperated side (NPL, NTS, and NIT). The SFI was calculated by the following formula derived by Bain et al [29]. An SFI of − 100 indicates total impairment, which could result from complete transaction of the sciatic nerve, whereas an SFI of 0 is considered a normal function (Figs. 3 and 4).

SFI formula:

$$ \mathrm{SFI}=\left(-38.3\times \mathrm{PLF}\right)+\left(109.5\times \mathrm{TSF}\right)+\left(13.3\times \mathrm{ITF}\right)-8.8 $$

$$ \mathrm{PLF}\ \frac{\mathrm{EPL}-\mathrm{NPL}}{\mathrm{NPL}};\mathrm{TSF}=\frac{\mathrm{ETS}-\mathrm{NTS}}{\mathrm{NTS}};\mathrm{ITF}=\frac{\mathrm{EIT}-\mathrm{NIT}}{\mathrm{NIT}} $$

### Histomorphometric examination

**Fig. 5:**
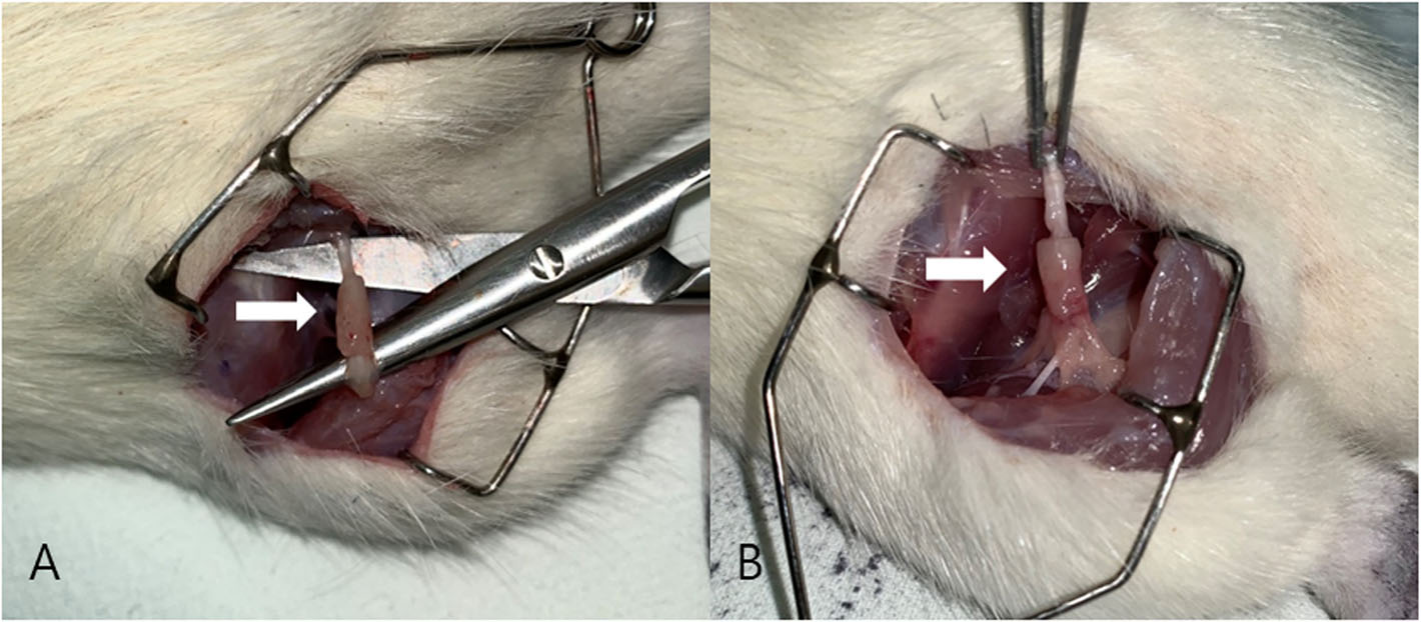
Harvesting the sciatic nerve. (**A**) Representative sciatic nerve of group C at 2 weeks. (**B**) Representative sciatic nerve of group C at 4 weeks. The collagen membrane is observed to be maintained at both periods (arrow)

**Fig. 6:**
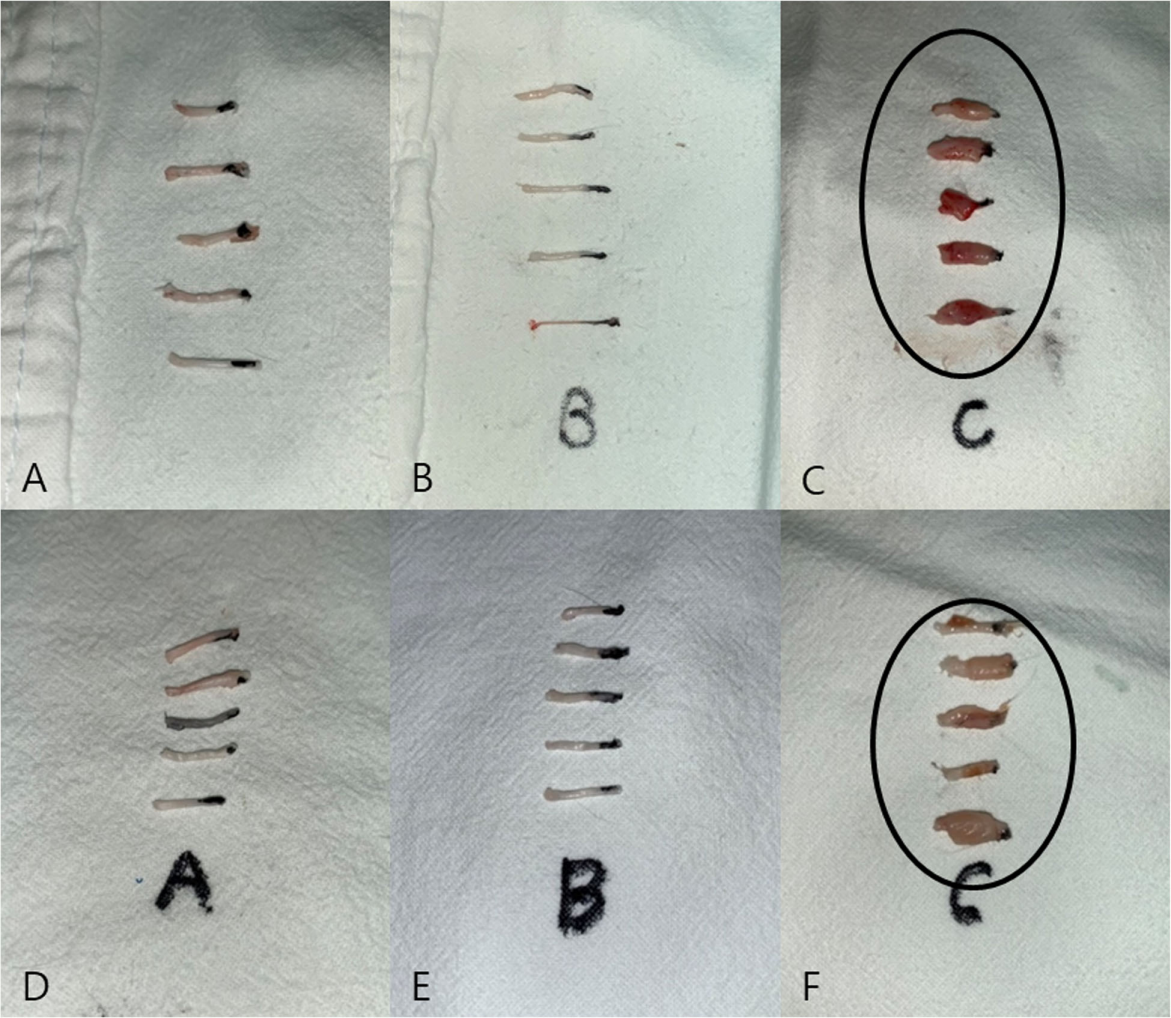
Sciatic nerve segments. All tissues were harvested with a length of 10 mm, and the proximal area was marked with black ink. **A** Nerve segments of group A at 2 weeks. **B** Nerve segments of group A at 4 weeks. **C** Nerve segments of group B at 2 weeks. **D** Nerve segments of group B at 4 weeks. **E** Nerve segments of group C at 2 weeks. **F** Nerve segments of group C at 4 weeks. The collagen membrane is demonstrated to be maintained at both periods (circle) in panels **C** and **F**

Shortly after the functional analysis, five randomly selected rats in each group were killed with CO_2_, and their sciatic nerve segments were harvested. Samples from each rat were fixed in 10% neutral formalin for 24 h, embedded in paraffin, and sectioned at 5 μm in thickness cross-sectionally and longitudinally. The sections were then stained with hematoxylin and eosin (H&E) and toluidine blue. Cross-sectional tissues (*n* = 4 for each group) were observed at a magnification of ×40 with a light microscope. Longitudinally sectioned tissues (one sample for each group) were examined at ×10 magnification. Toluidine blue–stained cross-sectional cuts were selected and examined under a light microscope. The total axon number and axonal density (axon number/mm^2^) were counted in the endoneurial areas using a randomized counting frame (Figs. 5 and 6).

### Statistical analysis

Experimental data (SFI and axonal density) are expressed as mean ± standard deviation. In this study, all measurements were based on multiple independent groups. The Kruskal-Wallis test was used to compare mean values of the three groups, and *p* < 0.05 was considered statistically significant. The Mann-Whitney test was used to compare the mean values of the groups two by two, and *p* < 0.017 (0.05/3: Bonferroni correction) was considered statistically significant. All statistical calculations were carried out using SPSS software (ver. 23.0, SPSS, Inc., Chicago, IL, USA).

## Results

### Nerve elongation and decrease in tension force during injury

Elongation of the sciatic nerve and tension force change at the start and end points of injury were measured during TNI (Table 1). The marked nerve length, which was initially 10 mm, increased to 11.9 ± 0.7 mm (range 11–13 mm) after injury. Further, the ratio of elongation was 18.5% ± 6.7% and the tension force decreased from 200 g to an average of 181.3 ± 5.1 g (range 175–190 g) in 1 min. Sciatic nerve rupture or discontinuity did not occur during the experiment.

**Table 1:** Measurement of nerve elongation and tension force change after tension injury

	Nerve length change			Tension force change		
	Original (mm)	After (mm)	Ratio (%)	Initial (g)	Final (g)	Ratio (%)
1	10	11.5	15	200	180	10
2	10	12	20	200	180	10
3	10	12	20	200	190	5
4	10	12.5	25	200	180	10
5	10	11.5	15	200	180	10
6	10	12	20	200	190	5
7	10	13	30	200	180	10
8	10	13	30	200	175	12.5
9	10	12.5	25	200	180	10
10	10	12	20	200	175	12.5
11	10	11.5	15	200	180	10
12	10	12	20	200	175	12.5
13	10	11	10	200	180	10
14	10	11.5	15	200	175	12.5
15	10	11	10	200	190	5
16	10	13	30	200	180	10
17	10	11	10	200	190	5
18	10	11	10	200	185	7.5
19	10	11.5	15	200	180	10
20	10	11.5	15	200	180	10
AV	10	11.9	18.5	200	181.3	9.4
SD	0	0.7	6.7	0	5.1	2.5

Original: original exposed nerve length, After: nerve length after tension injury, Initial: initial tension force, Final: tension at the time of tension removal

### Sole examination

All feet were inspected for signs of self-mutilation or injury before the walking track analysis. During the observation period, no corresponding manifestations were found in all animals (Fig. 3).

### SFI

**Fig. 7:**
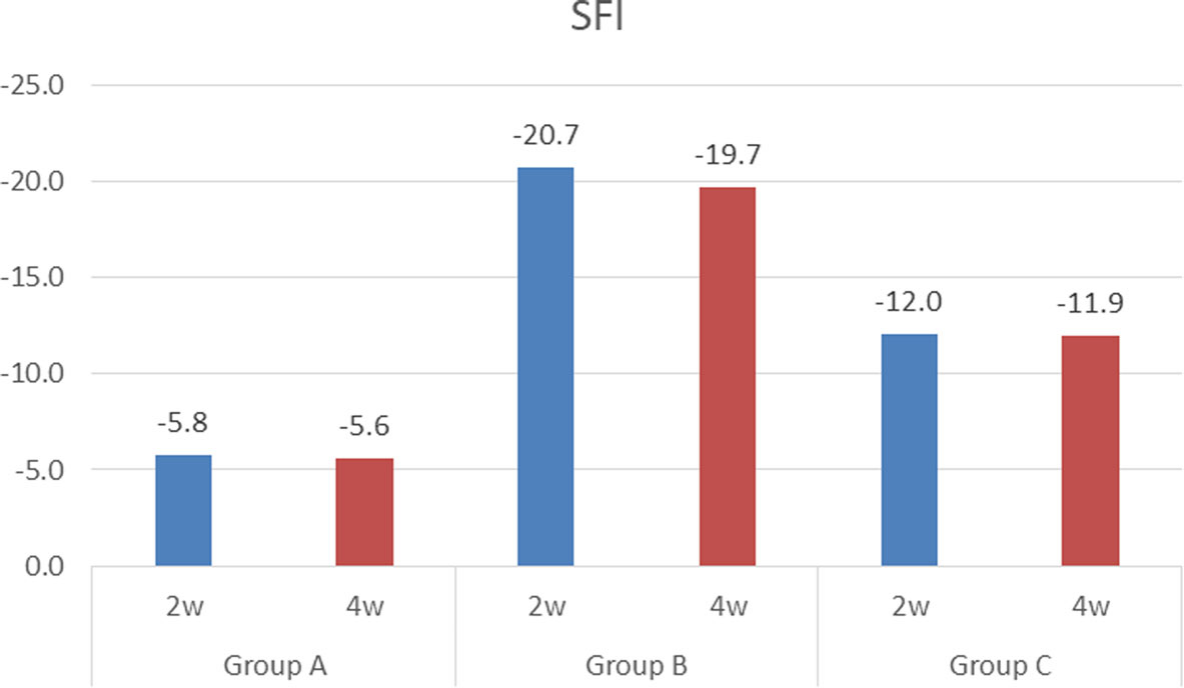
The mean value of the sciatic functional index in the three groups

The SFI was − 5.78 ± 3.07 for group A, − 20.69 ± 5.22 for group B, and − 12.01 ± 4.20 for group C at 2 weeks after surgery. After 4 weeks, the SFI was − 5.58 ± 2.45 for group A, − 19.69 ± 4.81 for group B, and − 12.01 ± 4.20 for group C (Table 2 and Fig. 7). Statistically significant differences were found in both periods for group A vs B, B vs C, and A vs C (*p* < 0.017) (Table 3).

**Table 2:** Sciatic function index

	Group A		Group B		Group C	
	2 weeks	4 weeks	2 weeks	4 weeks	2 weeks	4 weeks
1	− 9.9	− 8.0	− 15.1	− 29.6	− 7.6	− 13.2
2	− 9.0	− 4.6	− 19.0	− 18.3	− 13.2	− 13.0
3	− 1.2	− 7.0	− 21.3	− 19.7	− 13.2	− 9.6
4	− 8.8	− 5.7	− 18.7	− 17.9	− 17.7	− 12.4
5	− 8.8	− 7.6	− 18.9	− 15.1	− 15.8	− 11.9
6	− 9.2	− 7.5	− 25.4	− 21.1	− 11.1	− 13.9
7	− 6.8	− 8.0	− 15.5	− 17.6	− 15.6	− 13.8
8	− 9.9	− 8.6	− 16.3	− 16.4	− 14.5	− 9.0
9	− 8.3	− 4.2	− 13.7	− 17.7	− 14.7	− 6.9
10	− 9.0	− 8.4	− 24.9	− 15.3	− 8.1	− 14.8
11	− 7.2	− 7.2	− 24.3	− 17.8	− 3.8	− 13.6
12	− 8.0	− 8.5	− 17.5	− 15.7	− 6.9	− 9.8
13	− 6.1	− 7.9	− 17.4	− 19.4	− 13.3	− 10.9
14	− 7.1	− 7.3	− 17.6	− 19.7	− 13.7	− 11.8
15	− 4.5	− 7.7	− 16.4	− 17.6	− 18.5	− 9.5
16	− 1.7	− 6.5	− 30.7	− 21.7	− 15.8	− 13.7
17	− 8.0	− 5.3	− 24.4	− 19.7	− 12.2	− 11.3
18	− 0.4	− 5.0	− 20.9	− 24.9	− 15.0	− 12.2
19	− 6.4	− 2.1	− 14.0	− 17.3	− 14.6	− 12.2
20	− 8.5	− 7.4	− 30.5	− 16.8	− 14.2	− 13.2
21	− 4.6	− 3.3	− 24.8	− 24.7	− 7.9	− 13.0
22	− 7.8	− 4.2	− 17.3	− 15.4	− 3.9	− 11.5
23	− 1.5	− 8.1	− 25.4	− 25.6	− 8.1	− 12.4
24	− 4.8	− 8.3	− 14.4	− 24.6	− 3.8	− 11.9
25	− 5.7	− 5.9	− 26.3	− 24.1	− 6.9	− 13.9
26	− 6.8	− 5.8	− 19.2	− 32.4	− 13.3	− 13.7
27	− 8.4	− 0.3	− 21.8	− 16.9	− 13.7	− 9.4
28	− 6.8	− 6.5	− 24.6	− 15.7	− 18.5	− 8.2
29	− 1.2	− 1.0	− 24.1	− 15.1	− 15.8	− 15.0
30	− 7.4	− 2.7	− 32.4	− 15.9	− 12.2	− 13.6
31	− 0.3	− 6.2	− 16.9	− 22.2	− 15.0	− 9.8
32	− 0.6	− 5.7	− 15.7	− 17.9	− 11.1	− 10.9
33	− 2.5	− 0.9	− 15.1	− 20.0	− 15.6	− 11.8
34	− 4.5	− 0.4	− 24.4	− 28.4	− 14.5	− 10.0
35	− 1.7	− 1.5	− 20.9	− 11.7	− 14.7	− 16.3
36	− 8.0	− 4.8	− 14.0	− 13.7	− 8.1	− 11.3
37	− 0.4	− 5.7	− 30.5	− 17.1	− 3.8	− 12.1
38	− 6.4	− 6.8	− 24.8	− 13.7	− 6.9	− 12.2
39	− 8.5	− 3.9	− 17.3	− 27.1	− 13.3	− 12.6
40	− 4.6	− 6.8	− 15.5	− 26.2	− 13.7	− 11.7
AV	− 5.8	− 5.6	− 20.7	− 19.7	− 12.0	− 11.9
SD	3.1	2.4	5.2	4.8	4.2	1.9

**Table 3:** Statistical analysis of sciatic nerve function index

Group	*p*-value
2 weeks	
Group A vs Group B	0.001
Group B vs Group C	0.001
Group A vs Group C	0.001
4 weeks	
Group A vs Group B	0.001
Group B vs Group C	0.001
Group A vs Group C	0.001

*p* < 0.0167 (0.05/3: Bonferroni correction) was considered statistically significant

### Histologic analysis

**Fig. 8:**
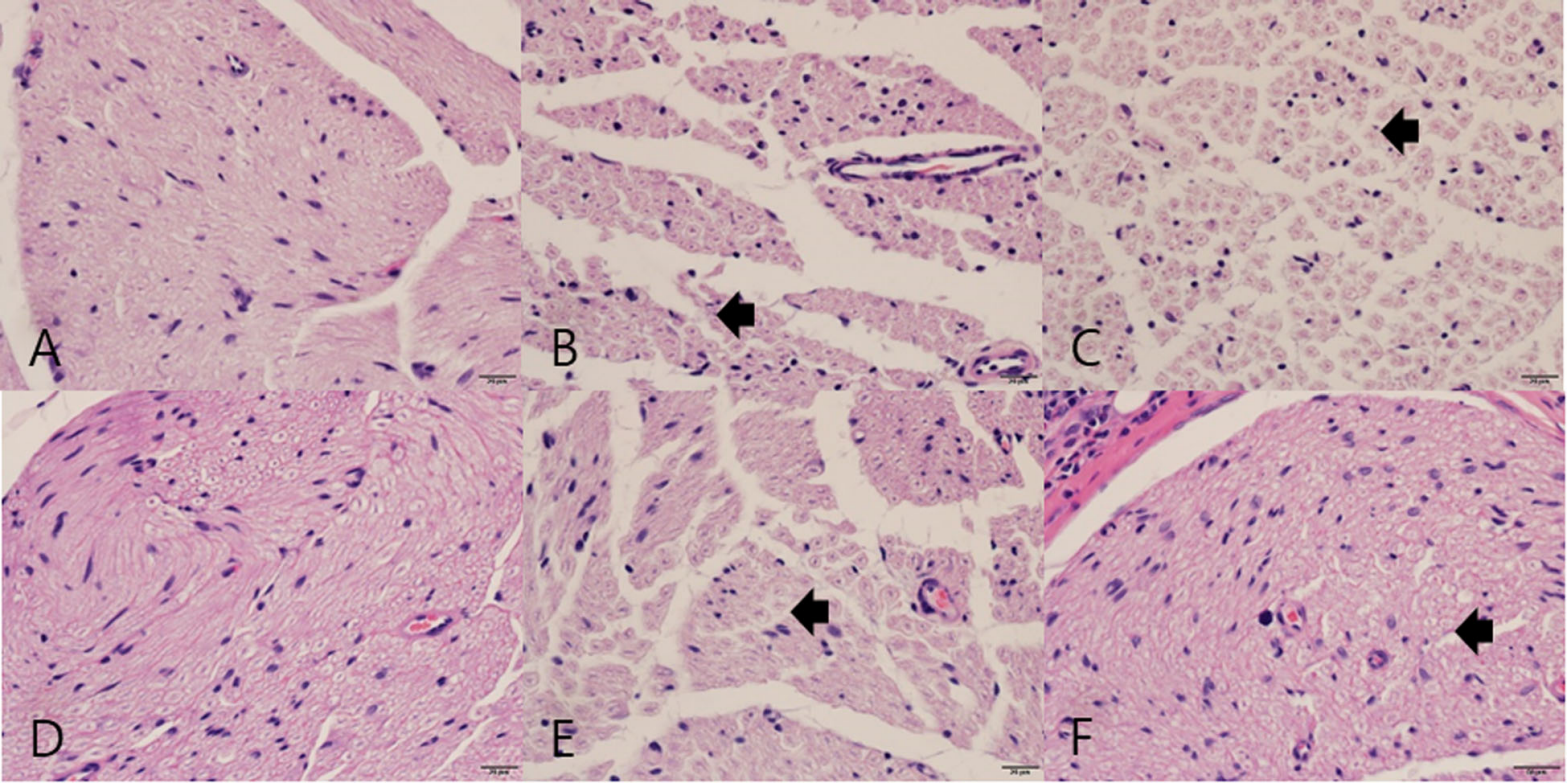
Cross-sectional image of the sciatic nerve. **A** Group A at 2 weeks: homogenous thickness and distribution of nerve fibers. **B** Group B at 2 weeks: dead spaces between fibers (arrow). **C** Group C at 2 weeks: separations between fibers (arrow). **D** Group A at 4 weeks: no significant difference with the result obtained at 2 weeks. **E** Group B at 4 weeks: dead spaces between fibers (arrow). **F** Group C at 4 weeks: decreased separations between fibers, with relatively homogeneous density compared with the result at 2 weeks after surgery (arrow). (Hematoxylin and eosin staining, ×40 magnification)

In the cross-sectioned microphotographs, homogenous thickness and distribution of nerve fiber (axons) were observed in group A, while irregularly spread nerve fibers and dead space between axons were observed in group B at 2 and 4 weeks after operation. In group C, separation between fibers was observed at 2 weeks after operation. However, this separation decreased at 4 weeks after surgery (Fig. 8).

**Fig. 9:**
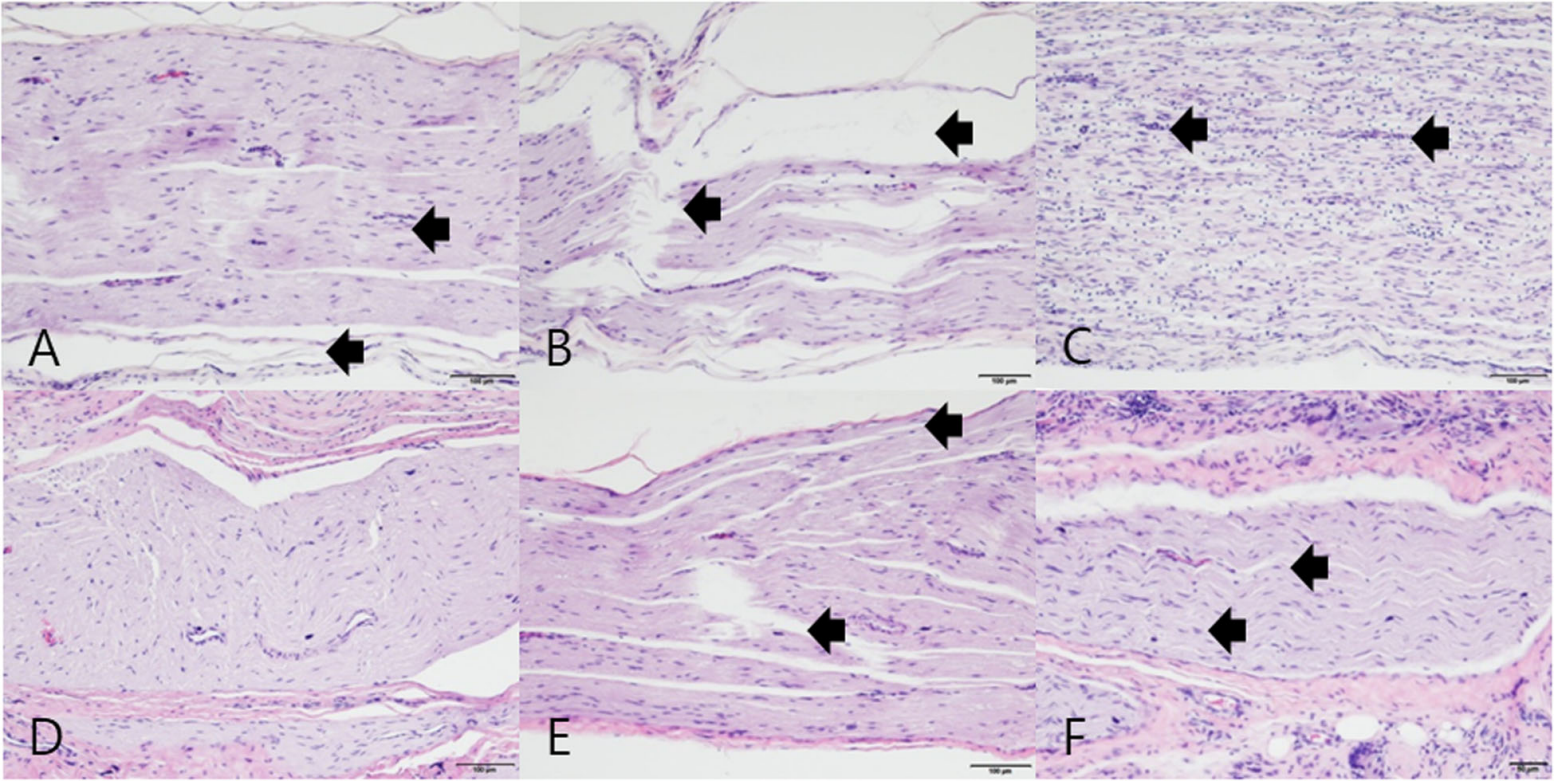
Longitudinal image of the sciatic nerve. **A** Group A at 2 weeks: orderly arranged fibers and intact epineurium (arrows). **B** Group B at 2 weeks: edema and severance (arrows). (**C**) Group C at 2 weeks: arrows indicate misarranged fibers with lymphocyte infiltration. (**D**) Group A at 4 weeks: no significant difference relative to the findings at 2 weeks. (**E**) Group B at 4 weeks: decreased edema and rupture relative to that previously observed (arrows). Well-defined curling and vacuolization are observed. (**F**) Group C at 4 weeks: orderly arranged fibers showing slight axonal swelling (arrows). (Hematoxylin and eosin staining, ×10 magnification)

In the longitudinal sections, clearly stained myelin sheaths were observed in group A, while nerve fibers with curling and vacuolization were observed in group B during the 2- and 4-week periods. In group C, tortuous fiber tracts and vacuolization were observed at 2 weeks after operation but were found to decrease at 4 weeks (Fig. 9).

**Fig. 10:**
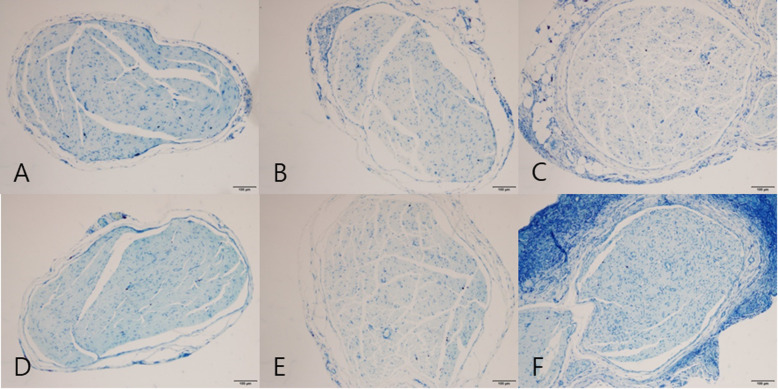
Cross-sectional image of the sciatic nerve in toluidine blue. **A** Group A at 2 weeks. **B** Group B at 2 weeks. **C** Group C at 2 weeks. **D** Group A at 4 weeks. **E** Group B at 4 weeks. **F** Group C at 4 weeks. (Toluidine blue staining, ×10 magnification)

In the cross-sectioned images of toluidine blue staining, axon number and axonal density were measured (Fig. 10). In both weeks, the axonal density in group B was lower than that found in other groups, and no statistically significant differences were found between group A vs B, B vs C, and A vs C (*p* < 0.017) (Table 4).

**Table 4:** Total number of axons and axonal density

	Cell count	Area (mm^2^)	Axonal density (axon/mm^2^)
Group (2 weeks)			
A	1954.25 ± 47.61	0.51	3862.51 ± 304.15
B	1510.75 ± 43.44	0.50	3022.41 ± 51.92
C	1742.75 ± 46.11	0.49	3539.57 ± 115.16
*p*-value between the groups		A and B	0.029
		B and C	0.029
		A and C	0.057
Group (4 weeks)			
A	1921.75 ± 25.06	0.51	3805.87 ± 32.52
B	1503.25 ± 21.17	0.50	3037.84 ± 58.44
C	1741.75 ± 36.18	0.50	3520.14 ± 103.24
*p*-value between the groups		A and B	0.029
		B and C	0.029
		A and C	0.029

*p* < 0.0167 (0.05/3: Bonferroni correction) was considered statistically significant

## Discussion

In this study, we sought to determine whether local FK506 delivery with collagen membrane and fibrin glue could improve nerve regeneration following TNI. By using a rat model, we analyzed the influence of FK506 using gait analysis and histologic findings. Based on the findings, local delivery of FK506 led to nerve regeneration outcomes.

Characterizing the thresholds for nerve damage under stretch is a compelling area in clinical and experimental applications. Several studies revealed that the minimum threshold for nerve stretch prior to functional deficit is between 5 and 10%. Jou et al. [30] demonstrated that rat femoral lengthening above 8% caused deficits in the sciatic nerve. Further, Rickett et al. [31] reported that after 10% stretch, the significant value of compound action potential amplitude decreased, indicating excess mechanical tolerance of the peripheral nerve. For disruption of the rat sciatic nerve, Spiegel et al. [32] revealed a mean of 626 g traction force. Fowler et al. [33] used 50 g of traction force, which continued for 5 min and resulted in significant functional deficit. Accordingly, 200 g of traction for 1 min was markedly sufficient for inducing functional deficit in this study. Further, the mean elongation rate (18.5%) surpassed the threshold reported in previous studies.

The SFI, which previously comprised four values (striding ability, the length of the footprint, spreading of the first and fifth toes, and spreading of the second and fourth toes), is considered a good assessment tool for overall nerve function in rat [34]. De Medinaceli et al. [35] reported that the components had equal importance. However, the formula of the index has been changed by several researchers [29, 36]. The striding ability has been excluded, and the remaining components have been assigned different weights according to their own statistical analysis. According to the index introduced by Bain et al. [29], which was used in our study, the values in normal gait and after complete transection of the sciatic nerve were compared. In our experiment, the index of the FK506 group was statistically different from that of the other groups after 2 and 4 weeks, highlighting the neurotrophic property. Although the SFI is an important measure for the recovery of the sciatic nerve function, comparison with other measures, such as histomorphometry or electrophysiology, should be performed to compensate for its limitations [37].

The prognosis of peripheral nerve repair depends on the extent of injury. Injury grading systems that correlate microscopic changes occurring after nerve injury with patient symptoms have been developed over several decades. The classifications of Seddon [38] and Sunderland [39] may serve as the most widely accepted ones. Seddon categorized the three types of injury as neurapraxia, axonotmesis, and neurotmesis [38], while Sunderland’s classification further grades the three injury types described by Seddon into five categories according to severity [39]. TNI usually results in first- or second-degree injury according to Sunderland’s classification. Correspondingly in our experiment, traction induced axonal injury while the connective tissue layers were preserved, indicating a second-degree injury. Separation and discontinuity of neighboring cells increased without perineural severance in the injury group. There was no significant difference among each group in axonal density based on statistical analysis; however, some degree of nerve regeneration was observed in histomorphometric examination. Histologically, the degree of nerve damage decreased at 4 weeks compared to that at 2 weeks in groups B and C. In addition, at 4 weeks, group C showed improved recovery relative to the injured group. Therefore, peripheral nerve injury caused by traction shows subsequent nerve regeneration, and FK506 might help to promote this process.

FK506 has been demonstrated to increase nerve regeneration by increasing the rate of axonal regeneration [21]. However, unlike the immunosuppressive mechanism, the exact mechanism of neuroregenerative effect of FK506 remains unclear [40]. Several studies have demonstrated that FK506 activity is mediated by a family of proteins, termed FK506-binding proteins (FKBP), and the 12-kDA receptor (FKBP-12) [41–43]. Researchers have shown that FKBP exists not only in T cells but also in neuronal tissues [44] and increases after axotomy. The complex of FK506 and FKBP-12 inhibits the calcium-activated phosphatase, calcineurin, increasing the phosphorylation levels of calcineurin substrates with growth-associated protein-43 (GAP-43) [45]. GAP-43, a calcineurin substrate in neurons, plays an important role in axon elongation [46] and growth cone formation [47]. Hence, FK506 could increase nerve regeneration by increasing the phosphorylation of GAP-43 before inhibiting calcineurin [22]. FKBP-52, also known as FKBP-59 or heat shock protein 56, was introduced with the neurotrophic properties of FK506 by Gold et al. [48]. FKBP-52 is a component of mature steroid receptor complexes [49], which associates with microtubules in the cytoplasm and nucleus [50], and plays an important role in cytoplasmic-nuclear shuttling of steroid receptors [51], which is decisive for neuronal growth. The present study revealed the clinical treatment potential of local FK506 in a peripheral nerve injury; however, detecting the molecular mechanisms of neurotrophic property remains to be elucidated.

Most studies that investigated the nerve regeneration effect of FK506 performed systemic administration. As a result, little is known about the localized delivery of FK506 to sites of nerve injuries [21, 24, 40, 52, 53]. In recent years, few studies have reported the neuroregenerative effect of locally delivered FK506. All studies tended to focus on the substances that control the emission of FK506. Azizi et al. [54] demonstrated the improvement of functional and morphometric recovery of rat sciatic nerve by loading FK506 in a vein graft. Mekaj et al. [55] applied FK506 by wrapping an absorbable gelatin sponge with a rabbit sciatic transection. Davis et al. used poly(l-lactide-ε-caprolactone) nerve wraps [56] and micro-patterned poly(lactic-co-glycolic acid) (PLGA) films [57] to deliver FK506 locally. However, each study had limitations, such as donor site morbidity, fast biodegradability, and high cost.

Among many biodegradable materials, collagen is favorable as a drug carrier. Highly purified type I collagen is processed into a tubular matrix with adequate mechanical strength and controlled permeability. OSSGUIDE®, the collagen membrane employed herein, is a highly purified type I collagen derived from porcine tissue and cross-linked membrane. The cross-linking process enhances the tensile strength of collagen and extends the degradation time [58]. This property is maintained in vivo for a long time during the healing period, which differentiates it from non-cross-linked membranes. Such characteristic was revealed in our experiment, as the collagen membrane slowly biodegraded and remained present when the nerve tissue was harvested after 2 and 4 weeks. Further, the semipermeable property of collagen conduits enables diffusion of neurotrophic factors from the external environment into the repair site [59].

Several experimental studies on the topical administration of FK506 after peripheral nerve injury have reported various doses. In vitro, up to 0.25 mg showed good results at the site in a rat sciatic nerve crush model [48]. In a rabbit model, Diaz et al. [25] suggested 10 ng/mL based on a comparison with a previous study [60] on corneal endothelium. Tajdaran et al. [27] reported a concentration of 200 μg based on previous studies [28, 61] that used a similar local dose of CsA. Taken together, the variety of dosage ranges for topical administration was associated with the drug’s persistence.

The particle form of FK506 may affect continuity of the drug’s effect. Tajdaran et al. [27] reported the effect of local delivery of FK506 incorporated into the fibrin glue by comparing forms of solubilized, particulate, and PLGA microsphere (MS) encapsulated FK506. All three forms were effective, but the particulate form and the MS encapsulated form showed superior axon regeneration. However, rather than their types, this experiment revealed the effectiveness of the fibrin glue–based delivery system relative to any conduit-based delivery in vivo.

Fibrin glue, which has been used in the surgical field for decades, is clinically easy to use. In addition, there is no need for a secondary surgery for the removal of the delivery system as it is biodegradable and biocompatible. Sameem et al. [62] reported that fibrin glue showed quicker and easier use modality than microsuture repair for a peripheral nerve injury. As a result, we used the 0.5 mg of solubilized form of FK506 with collagen membrane and fibrin glue. This method had advantages, such as persistency, clinical reproducibility, manipulability, and achievement of meaningful results.

FK506 has been reported to be associated with serious side effects related to dosing, including nephrotoxicity, hyperglycemia, and central nervous system toxicity [63]. The major side effect of FK506 is body weight loss accompanied by diarrhea [21]. Other symptoms include tremor, paresthesia, pain, and seizures. In our study, there were no clinical symptoms of body weight loss or self-mutilation. However, a detailed toxicological evaluation should be performed to ensure no systemic toxicity following the local administration of FK506.

In this study, we revealed the beneficial effects of local FK506 on nerve regeneration following TNI. Further, we demonstrated that collagen plays an important role as a permeable membrane while being safely maintained for a long period in vivo. As FK506 was used in a solubilized form, fibrin glue also appeared to help persistence and sustained local release. These results were obtained without any serious side effects. In the clinical situation of traction nerve injury, tacrolimus could be applied with collagen membrane and fibrin glue easily onto the injured nerve such as the mental, inferior alveolar, and facial nerves to promote early nerve regeneration. Of note, the molecular mechanisms of FK506 remain to be investigated for human experiment. The clinical application in humans could be possible if the toxicity and proper titer of tacrolimus are elucidated.

## Conclusion

Localized FK506 with collagen membrane and fibrin glue could promote axonal regeneration in a rat model of TNI.

## Data Availability

All the experimental data were retrieved from the animal experimental center of Asan Medical Center.

## Abbreviations

PNI: Peripheral nerve injuries
TNI: Traction nerve injury
CsA: Cyclosporine A
SD rats: Sprague-Dawley rats
SFI: Sciatic function index
(E or N) PL: (Experimental, normal) print length
TS: Toe-spreading
IT: Intermediary toe-spreading
H&E: Hematoxylin and eosin
FKBP: FK506-binding proteins
GAP-43: Growth-associated protein-43
PLGA films: Poly(lactic-co-glycolic acid) films
MS: Microsphere
